# Three new species of *Licania* (Chrysobalanaceae) from Peru

**DOI:** 10.3897/phytokeys.42.7924

**Published:** 2014-09-16

**Authors:** Ghillean T. Prance

**Affiliations:** 1 Royal Botanic Gardens, Kew, Richmond, Surrey, TW9 3AB, UK

**Keywords:** Chrysobalanaceae, *Licania*, Amazonian Peru

## Abstract

Recent collections received for identification contain three conspicuous new species for the mid altitude forests of Amazonian Peru. *Licania palcazuensis*, *Licania apiknae* and *Licania monteagudensis* are described as new and their relationship to other species is discussed. A key is provided for all the species of Licania subgenus Licania section Licania known to occur in Peru.

## Introduction

Since a world monograph of the Chrysobalanaceae (Prance and Sothers 2003a,b) new species are still being discovered (Prance 2013) and recently studied collections reveal three more in the genus *Licania*, all from Amazonian Peru, a region that is still yielding many novelties. The sterile inventory material that I have seen from this region indicates that there are many more yet to be described. The new species described here fall into two of the subgenera, subgenus *Licania* and subgenus *Moquilea*.

## Species descriptions

### Licania palcazuensis

Taxon classificationPlantaeMalpighialesChrysobalanaceae

Prance sp. nov.

urn:lsid:ipni.org:names:77142292-1

Figs 1 , 2

Ab omnibus speciebus Licaniae inflorescentibus multi-ramificantibus, pseudopedicellis 1–3 floribus instructis, pilis bracteolium glandulosis differt.

#### Description.

Tree to 25 m tall, young branches sparsely tomentellous, conspicuously lenticellate with age. Leaves with small triangular to linear stipules to 1.0 mm long, early caducous; petioles 4–7 mm long, terete, rugose, sparsely tomentellous; lamina oblong to oblong-lanceolate, subcoriaceous, 4–8.5 × 1.5–3 cm, cuneate at base, acuminate at apex, the acumen 4–8 mm long, glabrous above, densely rufous lanate-tomentose beneath, with scattered palisade glands mainly near to midrib; midrib plane above, prominent beneath; veins 10–12 pairs, plane above, prominulous beneath. Inflorescence terminal and axillary much-branched panicles with many short branches bearing 1–3 flowers, the rachis and branches rufous-brown tomentose. Bracts and bracteoles membraneous, triangular-acute, c 1 mm long, borne at base and on pseudopedicels, the ciliate margins with glandular hairs; pedicels 0–5 mm long, flowers articulate just below receptacle base where upper bracteoles are borne 2–7 mm below articulations. Receptacle campanulate, rufous-tomentose on exterior. Flowers seen only in young fruiting condition; calyx lobes 5, markedly triangular, tomentose on exterior, interior glabrous towards base, tomentellous towards apex. Petals triangular, margins ciliate. Stamens 12–14, inserted around complete circle, slightly exceeding calyx lobes in length. Style basal; ovary of young developing fruit densely rufous-tomentose becoming less so with age, unilocular with 2 ovules. Mature fruit not seen.

**Figure 1.**
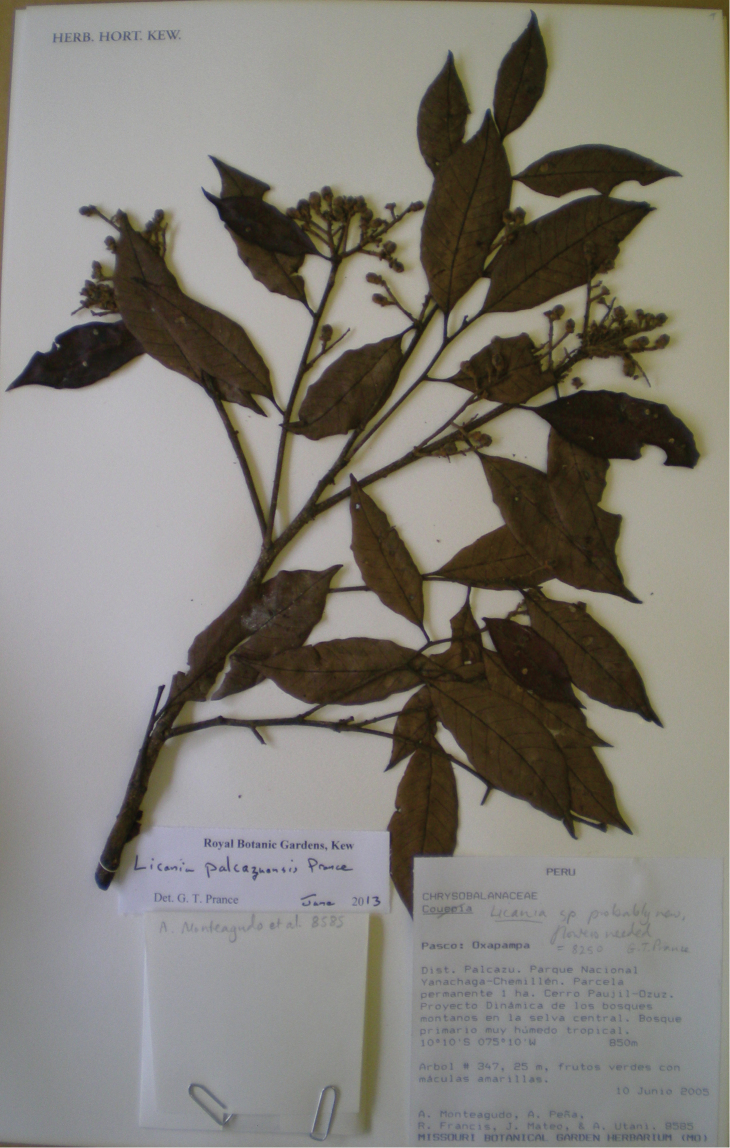
Photo of the holotype of *Licania palcazuensis* (Monteagudo et al. 8250).

**Figure 2.**
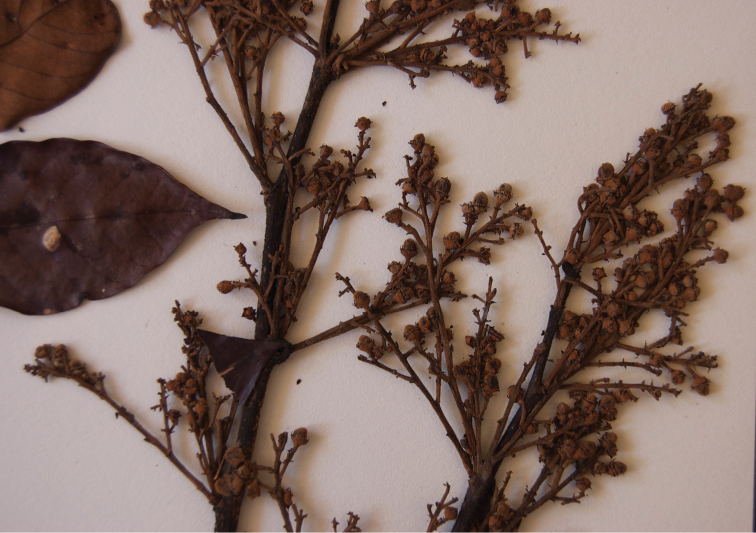
Close up of distinctive inflorescence of *Licania palcazuensis*.

Peru. Pasco: Oxapampa, Palcazu District, Parque Nacional Yanachaga-Chemillén, Cerro Panjil-Ozuz, Permanent plot tree 24, 10°10'S, 75°10'W, 850 m, 12 May 2005, *A Monteagudo, A Peña, R. Francis et al. 8250* (holotype, K; isotypes, AMAZ, HUT, MO, MOL, USM).

#### Additional material seen.

Same locality, Tree 347, 10 June 2005, 15 May 2005, *A. Monteagudo, A Peña, R. Francis et al. 8585* (AMAZ, HUT, K, MO, MOL, USM), Tree 171, 15 May 2005, *A. Monteagudo, A Peña, R. Francis et al. 8418* (K, MO USM).

Differs from all other species of *Licania* in the inflorescence branching with pseudopedicels bearing one or several flowers that articulate from it, and the bracteoles have marginal hairs that terminate in tiny glands. This species belongs to subgenus *Moquilea* on account of the number of exserted stamens and the presence of petals, but the inflorescence distinguishes it from all the other species of the subgenus. The field notes mention that the flowers are white and that the fruit has yellow spots.

### Licania apiknae

Taxon classificationPlantaeMalpighialesChrysobalanaceae

Prance sp. nov.

urn:lsid:ipni.org:names:77142293-1

Figs 3 , 4

Ab *Licania laxiflora* petiolis 10–13 mm longis (haud 4–8 mm), apicibus foliorum acutis haud acuminatis, floribus parvioribus differt.

#### Description.

Tree to 24 m tall, young branches glabrous, not lenticellate. Leaves with small lanceolate, caducous stipules to 2 mm long; petioles 10–13 mm long, sparsely puberulous or tomentellous when young, terete; lamina ovate-elliptic, coriaceous, 5–14 × 3.5–9 cm, rounded to subcuneate at base, acute to apiculate at apex, glabrous and shiny above; lower surface with deeply reticulate venation filled with a rufous pubescence; midrib impressed above, prominent beneath; veins 6–8 pairs, slightly impressed above, prominent beneath; secondary veins prominent and more or less parallel forming a reticulate pattern, tertiary venation flattened forming stomatal crypts. Inflorescence terminal and axillary towards apex of flowering branches, racemose once-branched panicles, the rachis and branches brown-tomentellous. Flowers ca 1.5–2 mm long, sessile on primary branches of inflorescence. Bracts and bracteoles minute, caducous. Receptacle cupuliform, sessile, short-tomentose on exterior, densely tomentose within; calyx lobes 5, acute, tomentose on both surfaces. Petals absent. Stamens 5–6, inserted to one side of ring. Style basal, pubescent for 2/3 of length, included; ovary pilose-tomentose, inserted at base of receptacle. Fruit not seen.

**Figure 3.**
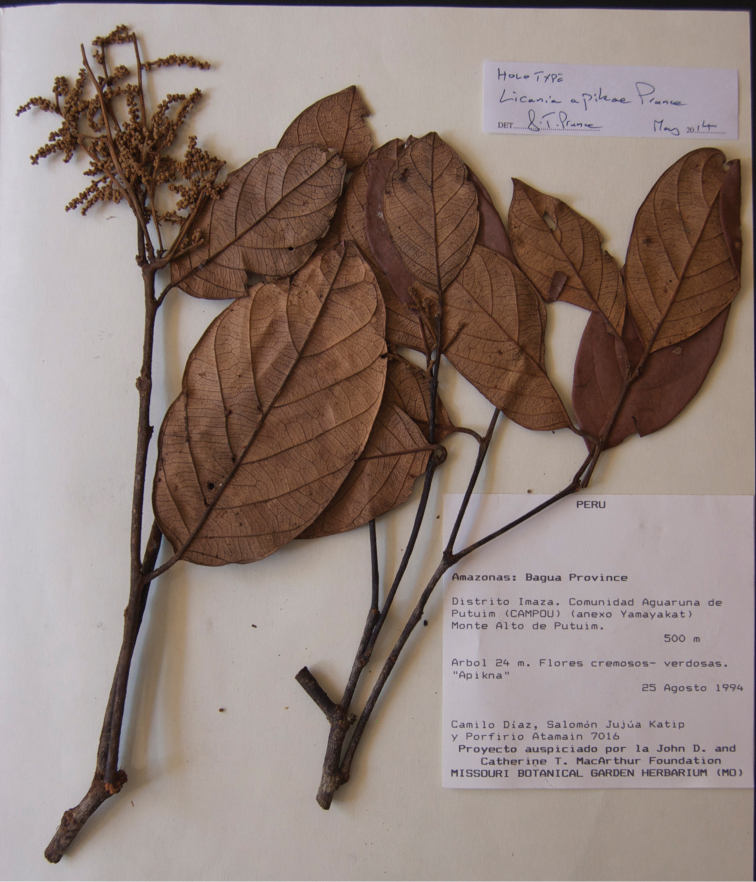
Photo of the holotype of *Licania apiknae* (Diaz et al. 7016).

**Figure 4.**
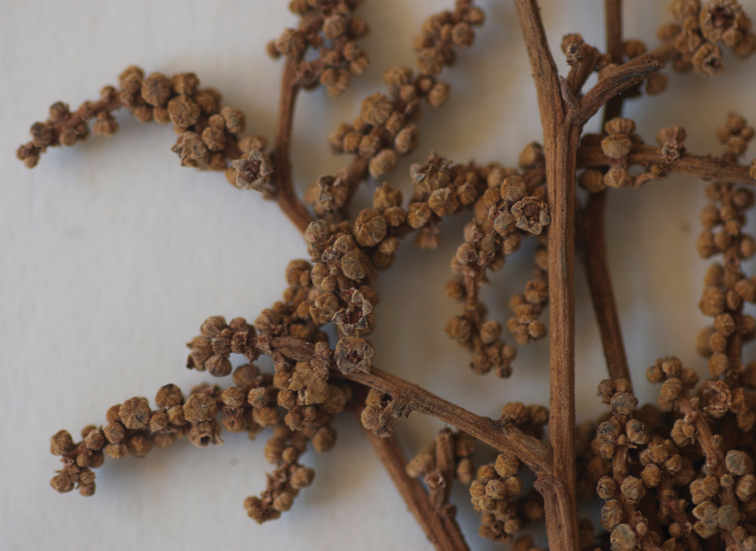
Close up of inflorescence of *Licania apiknae*.

Peru. Amazonas: Bagua Prov., Imaza Dist., Comunidad Aguaruna de Putuim, Monte Alto de Putuim, 5°00'54"S, 78°22'44"W, 500 m, 25 Aug 1994, *C.Diaz, S.J.Kayip, & P. Atamain 7016* (holotype K; isotype MO).

#### Additional material seen.

Peru. Amazonas: Bagua Prov., Imaza Dist., Comunidad de Yamayakat, Quebrada Kus-Chapi, Río Marañon, 04°55'S, 78°19'W, 550 m, Feb 1995, *R. Vásquez et al. 19477* (K, MO); Comunidad Aguaruna de Putuim, Monte Alto de Putuim, 22 Aug 1994, *C. Diaz et al. 7041* (K, MO).

Closest to *Licania laxiflora* Fritsch a species of the Guianas and Central Amazonia, but differs in the blunt leaf apex, the longer petioles (10–13 mm versus 4–8 mm), the smaller flowers and bracteoles and the more compact inflorescence. This species has been confused with *Licania harlingii* Prance, but differs from that species in the longer petioles (5–6 mm in *Licania harlingii*), the blunter leaf apex, the deeply reticulate leaf venation beneath. It belongs to subgenus *Licania* section *Licania* on account of the included stamens and the absence of petals. The name for this species is derived from “Apikna”, the Aguaruna name for it.

### Licania monteagudensis

Taxon classificationPlantaeMalpighialesChrysobalanaceae

Prance sp. nov.

urn:lsid:ipni.org:names:77142294-1

Figs 5 , 6

Ab *Licania harlingii* foliis coriaceis, minoribus 3–7 × 1.5–3.5 (haud 7–12 × 3–7 cm), venis 6–7 (haud 8–11), petiolis 2–3 mm longis (haud 5–6 mm) differt.

#### Description.

Tree to 25 m tall, the young branches sparsely puberulous, not conspicuously lenticellate. Leaves with lanceolate stipules to 2 mm long, caducous, adnate to base of petiole; petioles 2–3 mm long, terete, tomentellous when young; lamina elliptic, coriaceous, 3–7 × 1.5–3.5 cm, cuneate at base, acuminate at apex, the acumen 3–6 mm long, glabrous above, densely brown-tomentellous beneath; midrib plane above, prominent beneath; veins 6–7 pairs, plane above, prominulous beneath. Inflorescence of terminal and subterminal panicles of racemes, the rachis and branches yellow-brown tomentose. Bracts and bracteoles lanceolate to triangular, 1–2 mm long, tomentose, caducous. Flowers almost sessile on primary inflorescence branches. Receptacle turbinate, tomentose on exterior, densely tomentose--pilose within, constricted at base to a minute pedicel 0.5 mm long; calyx lobes 5, acute, triangular, tomentose on exterior, sparsely tomentose within. Petals absent. Stamens 5–6, inserted opposite four calyx lobes. Style basal, pubescent for ¾ of length; ovary rufous tomentose. Fruit pyriform, 2–2.5 × 1 cm, exterior densely rufous-brown tomentose.

**Figure 5.**
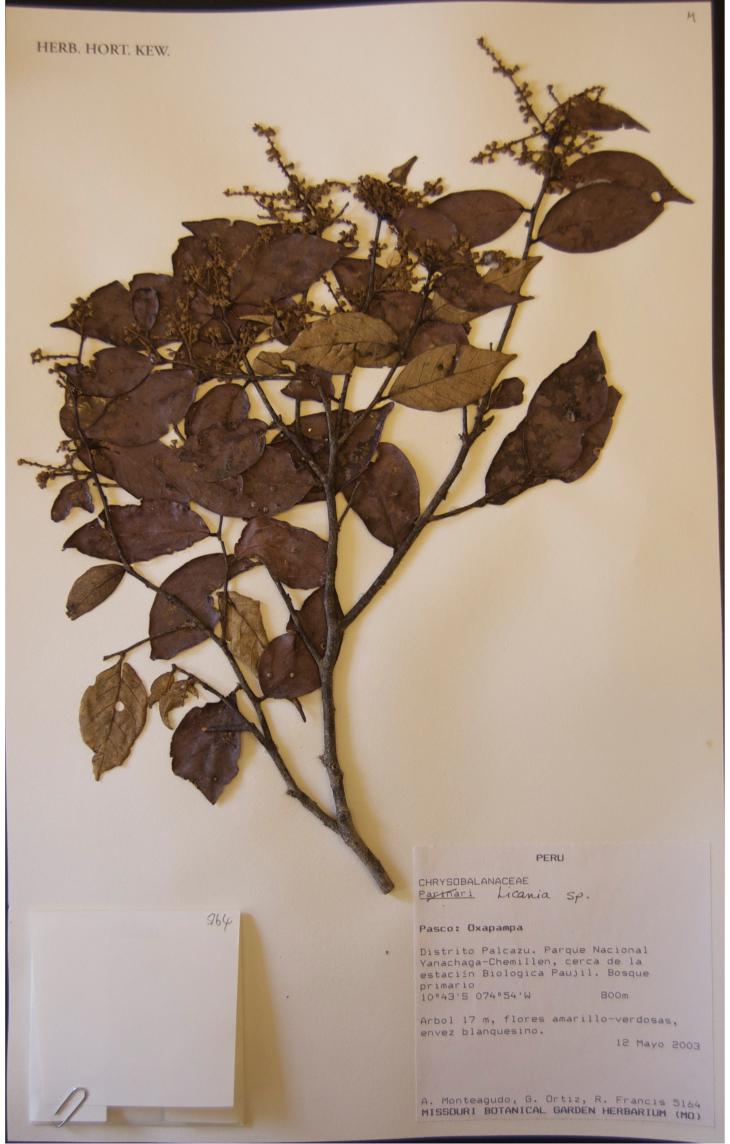
Photo of the type of *Licanis monteagudensis* (Monteagudo et al. 5164).

**Figure 6.**
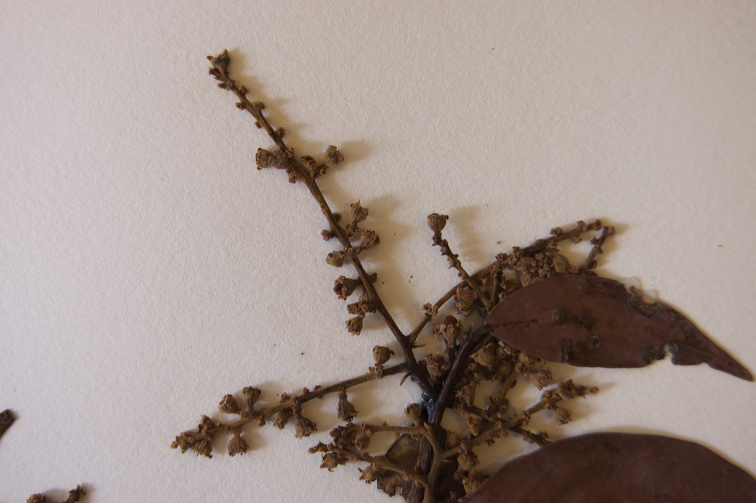
Close up of the inflorescence of *Licania monteagudensis*.

Peru. Pasco: Distr. Palcazu, Parque Nacional Yanachaga-Chemillén, Estación Biologica Paujil, 10°43'S, 74°54'W, 800 m, 12 May 2003, *A. Monteagudo, G. Ortiz & R. Francis 5164* (holotype, K; isotype, MO)

#### Additional material seen.

Ecuador. Morona-Santiago: Limon Indanza, Cordillera del Condór, Comunidad Shuar Warints, 03°09'16"S, 78°14'50"W, 1020 m, 5 Oct 2002, *G. Toasa 8917* (AAU, K, MO, NY, QCNE, US). Peru. Pasco: Distr. Palcazu, Parque Nacional Yanachaga-Chemillén, Cerro Paujil-Ozuz, 10°10'S, 75°10"W. 850 m, 15 May, 2005, A. *Monteagudo et al. 8418* (K, MO, USM). Amazonas: Bagua Prov., Distr. Imaza, Comunidad Aguaruna Putuim, Anexo de Yamayakat, SW of Putuim, 700–750 m, 20 Jan 1996, *C. Díaz et al. 7723A* (K, MO); Cerros de Putuim, 5°03'20"S, 78°20'23"W, 350 m, 12 Jun 1996, *R. Vásquez et al. 21114* (K, MO);Tyu Mujaji, Comunidad Wawas, 5°15'56"S, 78°22'07"W, 600 m, 25 Oct 1997, *R. Vásquez et al. 24699* (K, MO); Quebrada El Amendro, 5°14'40"S, 78°21'24"W, 430 m, 9 Mar 1998 (K, MO).

This species falls into subgenus *Licania* section *Licania* and it is close to *Licania harlingii* Prance but differs in the smaller more coriaceous leaves (3–7 × 1.5–3.5 vs 7–12 × 3–7 cm), fewer veins (6–7 vs 8–11) and the shorter leaf acumen. The habitat is noted as primary forest. This is named for Abel Monteagudo, the collector of the types of two of the species described here.

Since two of these new species and the recently described *Licania condoriensis* Prance (2013) from the borders of Peru and Ecuador all belong to subgenus *Licania* section *Licania* I have provided a key based mainly on vegetative characters to all species of the section known to occur in Peru. All other species of *Licania* from Peru fall into other subgenera and sections of *Licania* of Prance and Sothers (2003a). They differ from *Licania apiknae*, *Licania condoriensis* and *Licania monteagudensis* in one or more of the following characters:

Stamens 10–50, exserted (Subgenus *Moquilea*)

Petals present

Leaf undersurface with a furfuraceous pulverulent pubescence (Section *Pulverulenta*)

Inflorescence a panicle of cymules (Section *Cymosa*)

Leaf undersurface glabrous or with a hirsute pubescence (Sections *Hymenopus* and *Hirsuta*)

### Key to Peruvian species of Licania subgenus Licania section Licania

**Table:** 

1	Stipules adnate to base of petiole, usually persistent	
2	Leaf base usually subcordate, midrib impressed above	
3	Leaf undersurface with hair-filled stomatal crypts; stamens 5	***Licania bracteata* Prance**
3'	Leaf undersurface deeply reticulate, but without stomatal crypts; stamens 8–11	***Licania mollis* Benth**
2'	Leaf base rounded to cuneate, never subcordate; midrib plan or impressed	
4	Leaf lower surface with stomatal crypts	***Licania parviflora* Benth**
4'	Leaf lower surface deeply reticulate or plane under pubescence	
5	Leaf apex round or mucronate; midrib deeply impressed; primary veins 10–12 pairs	***Licania paraensis* Prance**
5'	Leaf apex acute or acuminate, midrib plane or slightly impressed; primary veins 5–9 pairs	
6	Leaf undersurface smooth under dense lanate-farinaceous pubescence	
7	Flowers 1.5–2 mm, flowers and inflorescence with sparse grey-puberulous pubescence not completely covering surface	***Licania kunthiana* Hook. f.**
7'	Flowers 2.5 mm, flowers and inflorescence with a dense tomentellous pubescence	
8	Leaves 4–15 × 2.5–8 cm; stamens 3	***Licania micrantha* Miq**
8'	Leaves 3–7 × 1.5–3.5 cm; stamens 5–6	***Licania monteagudensis* Prance**
6'	Leaf undersurface reticulate under pubescence which is hard to remove; flowers and inflorescence with densely tomentellous pubescence	
9	Petioles glabrous; leaf undersurface only slightly reticulate	***Licania cidii* Prance**
9'	Petioles tomentellous even when old; leaf undersurface deeply reticulate	***Licania blackii* Prance**
1'	Stipules axillary and often caducous	
10	Leaf base distinctly cordate, lamina triangular-ovate; midrib and petioles villous-pubescent	***Licania trigonioides* J. F. Macbr.**
10'	Leaf base rounded to cuneate; lamina usually elliptic; midrib and petioles glabrous or short-puberulous	
11	Leaf undersurface with stomatal crypts	
12	Stamens 3; primary leaf veins 7–9 pairs	***Licania triandra* Mart. ex Hook. f.**
12'	Stamens 5–8; primary leaf veins 13–15	***Licania condoriensis* Prance**
11'	Leaf undersurface without stomatal crypts (deeply reticulate in *Licania apiknae*)	
13	Petioles 10–13 mm long; leaf undersurface deeply reticulate; midrib impressed above	***Licania apiknae* Prance**
13'	Petioles 5–6 mm long; leaf undersurface more or less plane under pubescence; midrib plane or slightly impressed	***Licania harlingii* Prance**

## Supplementary Material

XML Treatment for Licania palcazuensis

XML Treatment for Licania apiknae

XML Treatment for Licania monteagudensis
